# Paris Agreement on Climate Change: A Booster to Enable Sustainable Global Development and Beyond

**DOI:** 10.3390/ijerph13111134

**Published:** 2016-11-14

**Authors:** Subhash Janardhan Bhore

**Affiliations:** Department of Biotechnology, Faculty of Applied Sciences, AIMST University, Bedong-Semeling Road, Bedong 08100, Kedah, Malaysia; subhash@aimst.edu.my or subhashbhore@gmail.com; Tel.: +60-4-429-8176

**Keywords:** climate change, environment, global sustainability, go green, greenhouse gas emission, poverty, pollution, public health, sustainable development goals, United Nations

## Abstract

The global warming and its adverse effects on the atmosphere, the biosphere, the lithosphere, and the hydrosphere are obvious. Based on this fact, the international community is fully convinced that we need to fix the problem urgently for our survival, good health, and wellbeing. The aim of this article is to promote the awareness about the United Nations (UN) historic ‘Paris Agreement on Climate Change (PACC)’ which entered into-force on 4 November 2016. The expected impact of PACC on the global average temperature rise by 2100 as well as its role in enabling accomplishment of global sustainable development goals (SDGs) for the people and planet is also highlighted.

It is indeed a great moment for the global community as the United Nations (UN) historic Paris Agreement on Climate Change (PACC) came into force on 4 November 2016 with jubilant celebrations. The international community had realized and was completely convinced that climate change is affecting global health, poverty, food security, and national and global security. This agreement is a clear indication that our global policy makers and stakeholders of global sustainable development are exceptionally determined to mitigate the inevitable climate disaster and accelerate sustainable development for the benefit of the people and the planet.

The main aim of the PACC is to keep the increase in global average temperature to well below 2 °C, and to 1.5 °C if possible; the main objective of it is to decrease greenhouse gas emissions significantly as soon as possible [1].

Increased greenhouse gas emissions and the rise in global temperature is damaging global climate, biodiversity, and ecosystems [1,2,3,4,5]. It is evident that climate change is also adversely affecting the global food supply chain [6,7,8], global public health, and global advancement as a whole [1]. Taking everything into account, greenhouse gas emissions and the rise in global temperature does directly affect the atmosphere, the biosphere, the lithosphere, and the hydrosphere [2,3,4]. Hence, it was necessary for the global community to come together and act together to deal with the challenge posed by climate change.

The predicted data suggest that global average temperature could increase by 4.8 °C by the end of 2100, and current policies (without PACC) were not good enough to deal with this global environmental issue [9]. Based on the facts, global leaders and stakeholders of global sustainability strongly believe that the PACC will help to reduce global greenhouse gas emissions and its adverse global impact. The threshold level is 2 °C (Figure 1); however, policy makers want to ensure that the global average temperature rise by 2100 will not exceed 1.5 °C by intensifying global efforts.

The 13th Sustainable Development Goal (SDG) among the 17 ambitious and challenging SDGs adopted by the UN member states is “(to) take urgent action to combat climate change and its impacts” [1]. In fact, the 13th SDG is directly and or indirectly linked with the other 16 SDGs. Therefore, it was necessary for the international community to take urgent action to mitigate climate change in order to facilitate unprecedented reductions in greenhouse gas emissions.

The PACC will serve as a gear in accomplishing SDGs (Figure 2). If all stakeholders of global sustainable development keep the same momentum for the achievement of SDGs, then we will certainly accomplish them in time; as a result, the PACC will help to improve the lives of people and to build a better and safer future with no one left behind. However, the fact is that we have limited time to achieve all global SDGs by 2030. Hence, the well-organized and effective implementation of the PACC by the UN member states and partners will ultimately determine the degree of success and its impact on the international community and global sustainability. In other words, the PACC has become an “international law” as a result of a very bold and ambitious step taken by the UN to facilitate global sustainable development and to make this planet a healthier and happier place to live.

Taken together, the PACC sets a course for all countries to limit global temperature increase by taking bold steps towards the reduction of greenhouse gas emissions to strengthen resilience with respect to the inevitable impacts of climate change. The enactment of the PACC marks the beginning of a new chapter for the global community and demonstrates that all countries around the world are serious about addressing climate change. There is no doubt that the landmark PACC will help not only in reducing greenhouse gas emissions at the global level but also in boosting global sustainable development. However, bearing in mind the massive task of accomplishing SDGs, there is a considerable amount of work that needs to be done.

## Figures and Tables

**Figure 1:**
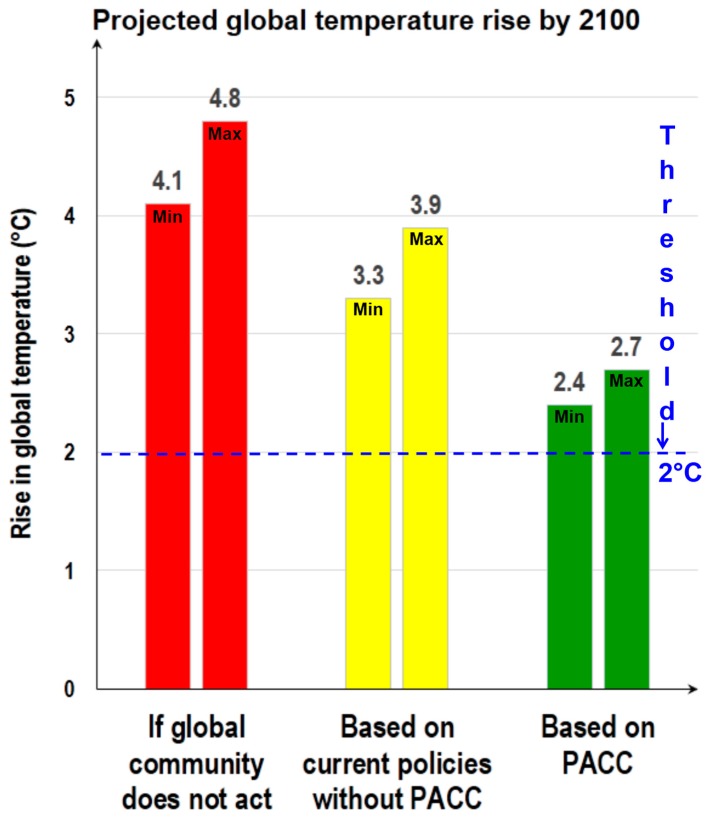
Projected global temperature rise by 2100 and the effect of current policies and the Paris Agreement on Climate Change (PACC). Min: minimum; Max: maximum. This figure is prepared based on the data published by Climate Action Tracker [9].

**Figure 2:**
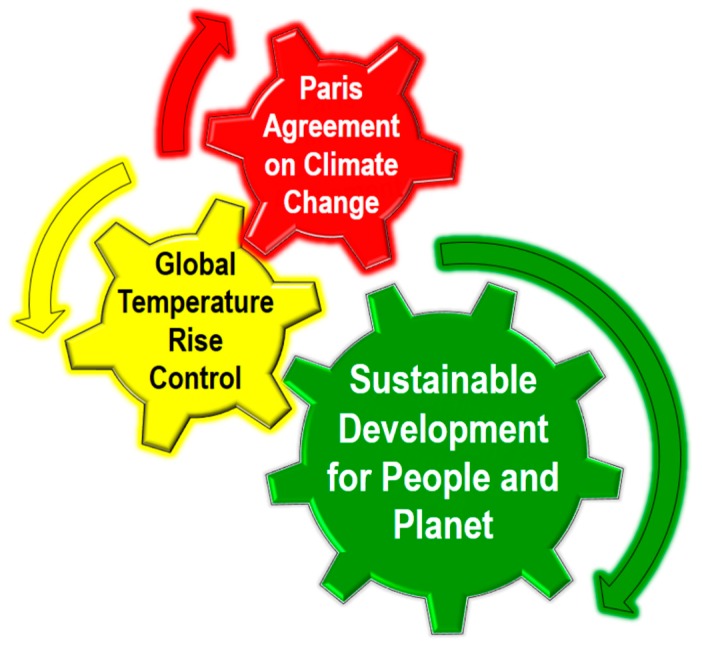
The Paris Agreement on Climate Change (PACC) will serve as a gear to control global temperature rise and to promote global sustainable development for the people and the planet.
